# Response to: A case of recurrent lentigo maligna diagnosed with precise reflectance confocal microscopy-guided biopsy technique

**DOI:** 10.1016/j.jdcr.2021.12.041

**Published:** 2022-01-21

**Authors:** Joanna Ludzik, Claudia Lee, Alexander Witkowski, Jonathon Hetts

**Affiliations:** a Department of Telemedicine and Bioinformatics, Jagiellonian University Medical College, Krakow, Poland; b Department of Dermatology, Oregon Health and Sciences University, Portland, Oregon; c School of Medicine, University of California Riverside, Riverside, California

**Keywords:** lentigo maligna, reflectance confocal microscopy, RCM, RCM-guided biopsy

*To the Editor:* We would like to commend Robinson et al^1^ for the introduction and successful application of a new reflectance confocal microscopy (RCM)-guided biopsy technique in the diagnosis of lentigo maligna (LM). LM is a common form of melanoma *in situ* that often shares many clinical features of benign pigmented lesions, making it diagnostically challenging for clinicians and pathologists. LM often demonstrates great heterogeneity, with areas displaying benign findings and other areas demonstrating atypical features, which increases the risk of sampling bias and misdiagnosis.^2^ Currently, guidelines recommend biopsies for LM be taken from the area with the highest concentration of pigment in order to minimize sampling error.^3^ Robinson et al^1^ proposed a clever solution by utilizing adhesive paper tape to delineate the area of the lesion with suspicious findings on RCM to guide biopsy. By creating a window for visualization in the tape, the lesion is evaluated through the window by handheld RCM (Vivascope 3000; CaliberID), constantly repositioning the tape until concerning RCM features are identified.^1^ The authors successfully implemented this tape technique to perform a precise RCM-guided biopsy in order to minimize sampling errors and improve diagnostic accuracy.

When evaluating large skin lesions, such as those typically seen in LM, the handheld RCM is often preferred over traditional RCM (Vivascope 1500, CaliberID) due to its rapid imaging and evaluation.^4^ The method by Robinson et al^1^ requires constant repositioning of the tape and limited visualization through a small window, which may be time consuming and pose a barrier to the widespread adoption of this technique, especially when evaluating large lesions. We propose an alternative method for performing RCM-guided biopsies that does not significantly increase the time needed for complete lesional assessment. With our proposed method, handheld RCM is used to evaluate the entire lesion rapidly and efficiently as part of standard assessment. If an area of the lesion is identified as concerning on RCM, a small drop of surgical ink is applied to the area of concern with a dip applicator to serve as a landmark for pathologists (Fig 1). The surgical ink is a sterile dye traditionally used to optimize intraoperative tissue orientation and margin marking.^5^ The ink securely adheres to tissue and brightly stains the area (Fig 2) for easy recognition by pathologists. Our technique for ink-stained RCM-guided biopsies is a simple, inexpensive, and less arduous method for optimizing lesion localization compared to what was proposed by Robinson et al.^1^ We have utilized this technique on several cases of RCM-identified suspicious pigmented and nonpigmented lesions, including a case of LM recurring in a scar. This highlights how the use of the described RCM-guided biopsy technique can be used to improve localization for biopsies for challenging cases and increase diagnostic accuracy in an efficient and time-sensitive manner that may have more widespread appeal compared to previously described techniques.

**Fig 1:**
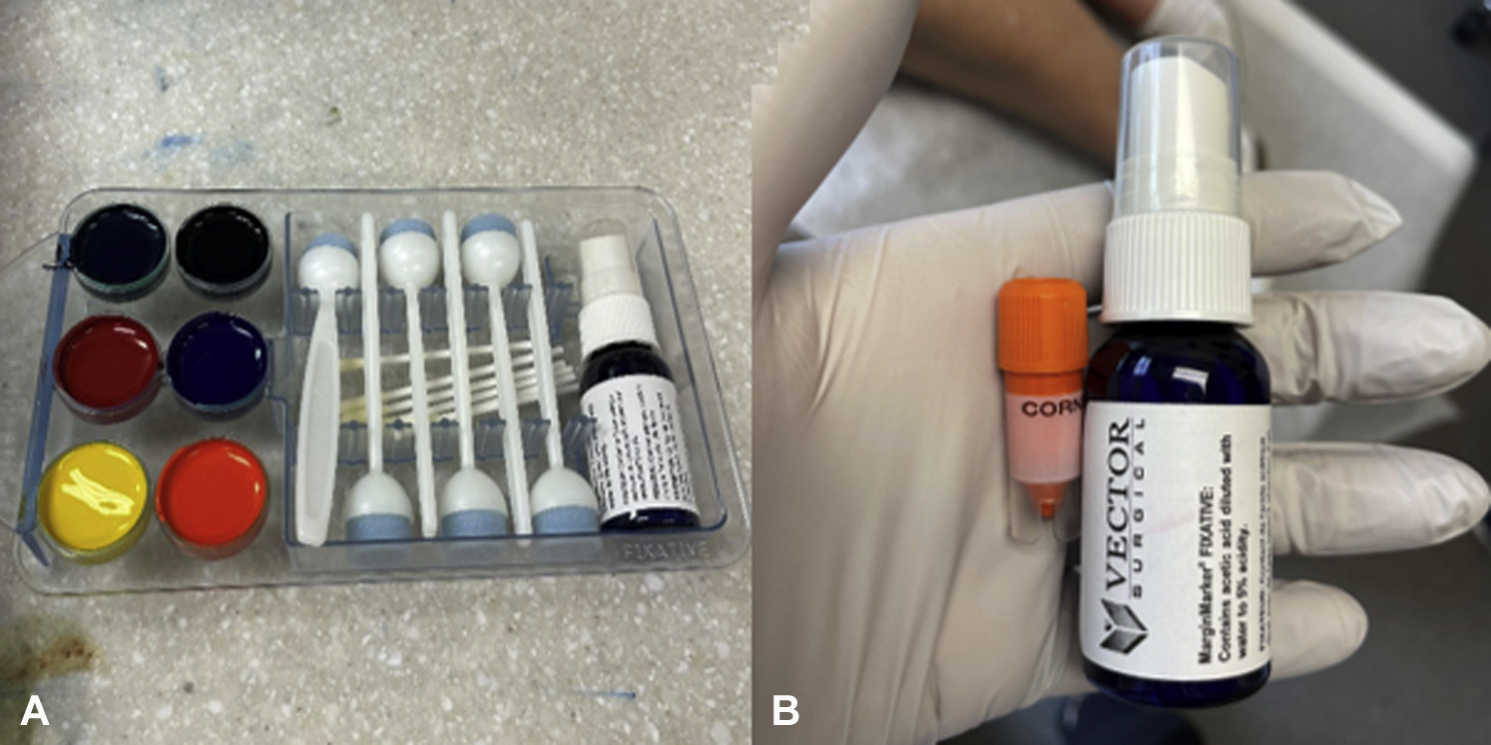
**A*,*** The ink kit (VectorSurgical MarginMarker) showing sterile dye with dip applicators. **B*,*** Fixative spray (VectorSurgical MarginMarker) containing acetic acid used to adhere the dye to the specimen.

**Fig 2:**
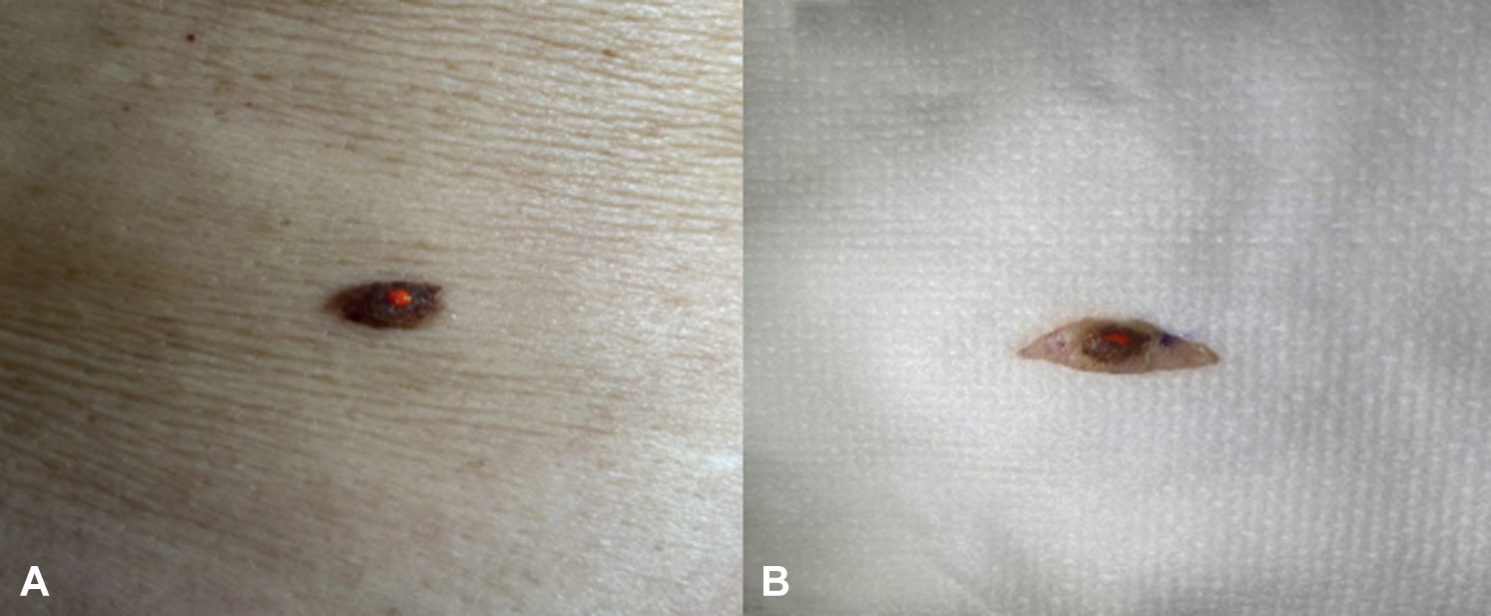
**A,** Clinical image of a pigmented lesion stained with surgical ink to demarcate the area of interest prior to excisional biopsy. **B,** Clinical image of the same ink-stained pigmented lesion postexcisional biopsy

## Conflicts of interest

None disclosed.
